# Complete chloroplast genome characterization and phylogenetic analysis of *Anredera cordifolia* (Tenore) Steenis (*Basellaceae*)

**DOI:** 10.1080/23802359.2021.1923427

**Published:** 2021-06-14

**Authors:** Zijing Weng, Shan Shen

**Affiliations:** a School of Basic Medical, Guizhou Medical University, Guiyang, China; b School of Life Sciences, Guizhou Normal University, Guiyang, China

**Keywords:** Chloroplast genome, *Anredera*, Madeira vine, phylogenetic analysis, liana, medicinal plant

## Abstract

Madeira vine (*Anredera cordifolia* (Tenore) Steenis) is a widely distributed liana that is also used as a medicinal plant. In this study, the complete chloroplast (cp) genome of this species was sequenced and annotated. The complete cp genome sequence of *A. cordifolia* is 156,662 base pairs in length, and the GC content is 36.84%. The genome contains 123 functional genes, including 83 protein-coding genes, 36 tRNA genes, and four rRNA genes. The phylogenetic tree showed that *A. cordifolia* was closely related to *Cistanthe longiscapa*, the species of family *Montiaceae* in *Caryophyllales*. This study provides genetic information for future studies on phylogenetic evaluation and utilization of *A. cordifolia*.

Madeira vine (*Anredera cordifolia* (Tenore) Steenis) is a kind of perennial, vigorous climbing liana with a twining and hairless stem. It belongs to the family *Basellaceae*, which includes approximately 25 species. Maderia vine is native to tropical and sub-tropical areas of South America, and is now widely distributed and extended to mild temperate climates worldwide (Vivian-Smith et al. 2007). It is classified as a problematic, noxious, or declared weed in some countries, for example, Australia, America, New Zealand, and South Africa (Vivian-Smith et al. 2007; Bari et al. 2019). However, Madeira vine is also known as a famous medicinal plant for health or as herbal medicine. According to Encyclopedia of Traditional Chinese Medicines, Madeira vine has the effect of tonifying the kidney, dispersing swelling and dissipating stasis. During convalescence, it can be used not only to treat fractures, knocks, falls, and weakness, but also to strengthen lumbus, relieve lumbus, and knee soreness (Zhou et al. 2016). In Indonesia, Madeira vine, known locally as Binahong, is known especially for healing wounds and treating several diseases, such as diabetes, hepatitis, and cardiovascular disease (Astuti et al. 2011). In addition, previous studies have also reported the pharmacological properties of Madeira vine leaf extracts in anti-hyperlipidemia (Lestari et al. 2015) and anti-hyperuricemia (Widyarini et al. 2015). Although Madeira vine has received much attention, its phylogeny has been poor studied. Herein, the complete chloroplast (cp) genome for Madeira vine was deciphered and the protein-coding genes were characterized.

Fresh leaves of Madeira vine were collected from the garden of Zunyi Hospital of Traditional Chinese Medicine in Guizhou Province, China (27°41′37.80″N, 106°55′52.60″E). The specimens were stored at Museum of Guizhou Normal University (https://www.gznu.edu.cn/, Qingbei Weng, wengqingbei@gznu.edu.cn) under the voucher number GZNU-WZJ-1. Total genomic DNA was extracted using a modified cetyltrimethylammonium bromide (CTAB) method (Doyle and Doyle 1987). The DNA libraries were constructed with an average length of 150 bp and then sequenced on Illumina HiseqXten (San Diego, CA). The Illumina reads were then fed into the NOVOPlasty (Dierckxsens et al. 2017) for *de novo* assembly. Since no cp genome of any species of family *Basellaceae* have been reported, the complete cp gene sequences of *Talinum paniculatum* (GenBank accession: MG710385.1), *Portulaca oleracea* (GenBank accession: KY490694.1) (Li et al. 2020), and *Cistanthe longiscapa* (GenBank accession: KX928992.1) of three species of order *Caryophyllales* were used as reference genes. For further evaluation and refinement, BWA-MEM (Li 2014) was used to map Illumina reads back to assembly. The cp genome was annotated by GeSeq 1.82 (Tillich et al. 2017), and tRNA genes were predicted by ARAGORN v1.2.38. The complete cp genome was deposited to GenBank with an accession number MW582603.

The complete cp genome of Madeira vine is a circular molecule of 156,662 base pairs (bp) in length, composing of a large single copy (LSC) region of 86,483 bp and a small single copy (SSC) region of 18,547 bp, respectively, which is separated by a pair of inverted repeat (IR) regions of 25,816 bp. The GC content of Madeira vine cp genome is 36.84%, and the corresponding values in LSC, SSC, and IR regions are 34.72%, 30.36%, and 42.72%, respectively. Totally, the cp genome was annotated with 123 genes, including 83 protein-coding genes (15 genes with two copies, five genes with three copies, and one gene with five copies), 36 tRNA genes, and four rRNA genes. The lengths of tRNAs range from 36 bp to 93 bp. The lengths of 4.5S rRNA, 5S rRNA, 16S rRNA, and 23S rRNA are 103 bp, 121 bp, 1491 bp, and 2811 bp, respectively.

To decipher the phylogenetic position of Madeira vine, a phylogenomic analysis were performed. The cp genome sequences of 17 species of order *Caryophyllales* were downloaded from GenBank, and the phylogenetic tree was constructed with the whole cp sequence by maximum-likelihood method using Mega X with 1000 bootstrap replicates (Kumar et al. 2018). The phylogenetic tree showed that compared to the species of other family in *Caryophyllales*, *A. cordifolia* has more closely related to *Cistanthe longiscapa* (*Montiaceae*), with a 100% bootstrap value (Figure 1). This study will provide useful genomic resources for further study on the species’ phylogeny, genetic variations, and designing utilization strategy.

**Figure 1.**
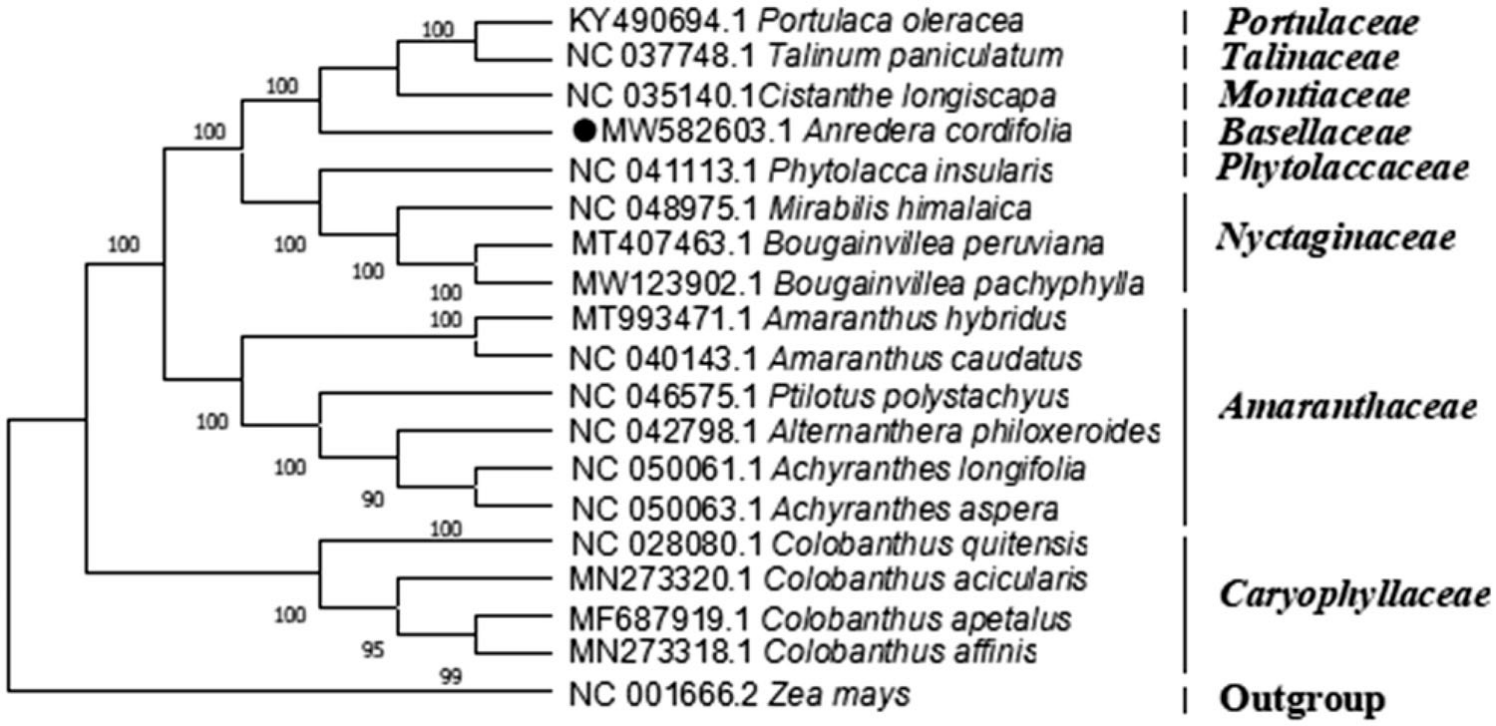
Phylogenetic position of *Anredera cordifolia*. The phylogenetic tree was generated by maximum-likelihood analysis based on the complete chloroplast genome sequences of 19 plants. *Zea mays* was selected as representative of the outgroup. The bootstrap values with 1000 replicates were used to confirm the stability of each tree node and were shown next to the node. GenBank accession numbers are listed left to the scientific names.

## Data Availability

The data that support the findings of this study are openly available in *Anredera cordifolia* (Tenore) Steenis at https://www.ncbi.nlm.nih.gov, accession number MW582603, Bioproject PRJNA694826, SRA SRP304753, and Biosample SAMN17722204. The data that support the findings of this study are available from the corresponding author, upon reasonable request.
